# Comparative ploidy response to experimental hydrogen peroxide exposure in Atlantic salmon (*Salmo salar*)

**DOI:** 10.1016/j.fsi.2018.07.017

**Published:** 2018-10

**Authors:** Lynn Chalmers, Luisa M. Vera, John F. Taylor, Alexandra Adams, Herve Migaud

**Affiliations:** Institute of Aquaculture, University of Stirling, Stirling, FK9 4LA, UK

**Keywords:** Triploid, Atlantic salmon, H_2_O_2_, Stress, Gene expression, Immune response

## Abstract

While research into the growth, survival, nutrition and, more recently, disease susceptibility of triploid Atlantic salmon has expanded, there remains an overall lack of studies assessing the response of triploids to chemical treatments. It is essential that the response of triploids to disease treatments be characterised to validate their suitability for commercial production. This study aimed to investigate and compare the stress and immune responses of triploid and diploid Atlantic salmon following an experimental treatment with hydrogen peroxide (H_2_O_2_). A dose response test was first undertaken to determine a suitable test dose for both diploid and triploid Atlantic salmon. Following this, diploids and triploids were exposed to H_2_O_2_ (1800 ppm) for 20 min, as per commercial practices, after which blood glucose and lactate, and plasma cortisol and lysozyme were measured, along with the expression of oxidative stress and immune-related genes. In the first 6 h post-exposure to H_2_O_2_, comparable mortalities occurred in both diploid and triploid Atlantic salmon. Cortisol, glucose and lactate were not significantly influenced by ploidy suggesting that, physiologically, triploid Atlantic salmon are able to cope with the stress associated with H_2_O_2_ exposure as well as their diploid counterparts. Exposure to H_2_O_2_ significantly elevated the expression of *cat* and *sod2* in diploid livers and *gr*, *il1β* and *crp/sap1b* in diploid gills, while it significantly decreased the expression of *saa5* and *crp/sap1a* in diploid gills. In triploids, the expression levels of *cat*, *hsp70*, *sod1*, *saa5*, *crp/sap1a* and *crp/sap1b* in liver was significantly higher in fish exposed to H_2_O_2_ compared to control fish. The expression of *gr*, *sod1* and *il1β* in triploid gills was also elevated in response to H_2_O_2_ exposure. This study represents the first experimental evidence of the effects of H_2_O_2_ exposure on triploid Atlantic salmon and continues to support their application into commercial production.

## Highlights

• This study represents the first testing of H_2_O_2_ in triploid Atlantic salmon.
• Following H_2_O_2_ exposure, triploid and diploid Atlantic salmon showed similar levels of cortisol, glucose and lactate.
• Ploidy differences in gene expression may be linked to extra genetic material in triploid cells, but requires further study.

## 1 Introduction

As the global population continues to grow, so will the demand for aquaculture food products, with the industry aiming towards the intensification and expansion of production [1,2]. Increasing intensification, however, is not without risks as it is considered that fish in intensive aquaculture systems can experience higher pathogen infection pressures than their wild counterparts [3,4]. As a result, disease and resultant health and welfare issues are recognised as one of the largest single causes of economic losses for aquaculture, representing a significant constraint to the industry's continued development and success [[5], [6], [7]].

Over the years, bacterial and viral diseases have caused significant problems for the Atlantic salmon (*Salmo salar*) aquaculture industry including those caused by *Aeromonas salmonicida*, *Moritella viscosa* and *Flavobacterium psychrophilum*; infectious pancreatic necrosis virus (IPNV), salmon alphavirus (SAV) and infectious salmon anaemia virus (ISAV) [8]. Effective vaccines have been developed to prevent and control many of these diseases, particularly for those caused by bacterial pathogens [6,[8], [9], [10]]. Currently, the issues associated with parasitic diseases pose the most significant threat for Atlantic salmon aquaculture. In particular, sea lice (*Lepeophtheirus salmonis*; *Caligus elongatus*) and, more recently, amoebic gill disease (AGD; *Neoparamoeba perurans*) are considered two of the most damaging parasites for the salmonid industry, with losses equating up to 430 million and 80 million USD ($) worldwide per year, respectively [[11], [12], [13], [14]]. With no vaccine available for the prevention of these parasites, a large proportion of the associated economic losses can be attributed to chemotherapeutic treatments [13]. One such treatment, now regularly employed by the aquaculture industry for the control of both sea lice and AGD, is hydrogen peroxide (H_2_O_2_) [15,16].

Hydrogen peroxide has long been used in aquaculture as a disinfectant for eggs [17]. Its use in the control for sea lice infections began in the early 1990's and it has been implemented to control AGD since 2012 [16,18]. In current aquaculture practices, the recommended H_2_O_2_ concentration for the treatment of these parasites is 1500 ppm although it is recognised that concentrations above 2000 ppm are used [[19], [20], [21]]. Several factors make this product suitable for application in aquaculture. First of all, it has a highly reactive nature which makes it ideal for combatting external parasites [17]. Hydrogen peroxide is a strong oxidising agent that causes mechanical paralysis, peroxidation of lipid and cellular membranes, inactivation of enzymes and inhibition of DNA replication. In sea lice, this compound appears to induce mechanical paralysis when bubbles form in the gut and haemolymph, causing the parasite to release from the host and float to the surface [22]. Additionally, when in the aquatic environment H_2_O_2_ breaks down quickly (1–10 days) into water and oxygen, therefore leaving no toxic by-products and making it reasonably environmentally friendly [17,23,24]. However, concerns have been raised regarding fish welfare during exposure to this chemical and it has been reported to cause stress in Atlantic salmon in the first 24 h post-exposure [25,26].

Triploid Atlantic salmon have long been considered as a solution to address production issues associated with pre-harvest sexual maturation and escapees in the aquaculture industry [27]. While many similarities with diploid salmon have been reported, differences have also been documented, with variable growth and increased deformities reported in triploids [[28], [29], [30], [31]]. With the expansion of triploid research over the last ten years, advances in triploid nutrition and rearing have now shown triploids performing equally or, in many cases, better than their diploid counterparts [[32], [33], [34], [35], [36], [37], [38]]. While recent research has continued to elucidate the response of triploid Atlantic salmon to disease [[39], [40], [41], [42], [43]], their response to disease treatments is still a relatively unexplored subject, particularly relating to chemical treatments such as H_2_O_2_. This is an important milestone given the increased environmental sensitivity reported in triploids when exposed to elevated temperatures and reduced oxygen levels [44,45]. Considering the potential to apply triploid salmon in full commercial production, it is crucial to understand their physiological response when exposed to aquaculture medicines in order to optimise health management strategies without compromising fish welfare. The aim of this study was to investigate the response of diploid and triploid Atlantic salmon to experimental exposure with H_2_O_2_ and assess susceptibility along with stress, immune and toxicological responses.

## 2 Materials and methods

### 2.1 Ethical approval

Experimental procedures were approved by the Animal Welfare and Ethical Review Body (AWERB) of the University of Stirling and were completed under UK Government Home Office project licence 60/4522. The euthanisation of fish for sampling was carried out according to the UK Government Home Office Schedule 1 regulations.

### 2.2 Fish stock and history

Eggs and milt were stripped from commercial Atlantic salmon broodstock (Landcatch Ltd.) and delivered to the Institute of Aquaculture, University of Stirling in December 2014. Following fertilisation, half of each egg batch was subjected to a pressure shock (655 bar for 6.25 min, 37.5 min post-fertilisation at 8 °C) to induce triploidy. Eggs were then incubated at 8.0 ± 0.1 °C in troughs until hatching (5th February 2015). At first feeding (2nd April 2015, 949 °D), fry were transferred into 300 L tanks and reared under constant light. Fry were fed a commercial diet (diploids - Inicio Plus; triploids – Inicio-TriX, BioMar UK), distributed by automatic feeders (Arvo-Tec Oy, Finland). When reaching 1 g (1738 °D), all fry were transferred to the Institute of Aquaculture freshwater unit at Buckieburn. They were maintained in 1.6 m^3^ tanks (<30 kg per m^3^) under ambient water temperature (average: 8.3 ± 4.2 °C; range: winter 1.5 °C – summer 14.0 °C) and photoperiod to produce S1+ smolts. Specific feeding rates (% tank biomass per day) were adjusted automatically according to predicted growth (verified by sample weigh every 6 weeks) and daily temperature, and pellet size (0.5–3.0 mm) increased with fish size. To verify ploidy status in each stock, smears were prepared from blood collected by tail ablation from euthanised fish at 5 g (100/ploidy). After air drying, slides were fixed in 100% methanol and then placed into 6% Giemsa stain (6 ml Giemsa in 94 ml distilled water) for 10 min. Erythrocyte length and diameter were measured at 40 × magnification using image capture software (Image-Pro Premier, MediaCybernetics, Rockville, USA). All erythrocytes were numbered then selected using a random number generator. A total of 20 randomly chosen nuclei per slide were measured to the nearest 0.01 μm. Diploid control groups had significantly smaller erythrocyte nuclear lengths (two-sided T-test, p < 0.05) with no overlaps with the pressure shock triploid groups (2 N 6.8–7.7 μm; 3 N 9.0–10.2 μm) confirming that fish subjected to hydrostatic pressure shock were triploids.

Diploid and triploid Atlantic salmon were then transferred to the Institute of Aquaculture Temperate Aquarium Facilities on 18th February 2016. In preparation for sea water transfer, fish were vaccinated on 14th March 2016 with WINVIL^®^ 3 micro (*Aeromonas salmonicida* subsp. *salmonicida*, *Moritella viscosa* & Infectious Pancreatic Necrosis Virus; Elanco Europe Ltd., United Kingdom). Mortality between first feeding and sea transfer was 4.8% and 5.1% for diploids and triploids, respectively. On 14th April 2016, 250 diploid (88.5 ± 2.2 g average body weight) and 250 triploid (78.2 ± 1.0 g average body weight) Atlantic salmon smolts were transferred to the Institute of Aquaculture seawater facilities at Machrihanish and stocked into two 2 m diameter stock tanks (3 m^3^; 0.5 L kg biomass^−1^ min^−1^ flow rate). Tanks were maintained under ambient temperature (11.5 ± 1.8 °C) with aeration provided by air stones for 96 days until the trial commenced.

### 2.3 Hydrogen peroxide stress challenge

#### 2.3.1 Dose-response toxicity test

A dose response test was first undertaken to determine an appropriate H_2_O_2_ dose for the stress challenge in diploid and triploid Atlantic salmon. This test was performed in July 2016 at ambient water temperature (14.0 °C) and simulated natural photoperiod set at 17 h light: 7 h darkness. For the dose response test, 21 diploid (183.0 ± 5.6 g body weight and 266.7 ± 2.4 mm body length) and 21 triploid (215.0 ± 4.9 g body weight and 282.4 ± 2.2 mm body length) Atlantic salmon were used. Experimental fish were randomly allocated into 14 × 397 L cylindrical tanks (n = 3 fish tank^−1^; 7 tanks ploidy^−1^). Following stocking, 1 tank ploidy^−1^ was allocated to each test concentration (1500, 1700, 1900, 2100, 2300, 2500 and 2700 ppm). Each concentration was then assessed separately. Fish were exposed to each concentration for 20 min before the tanks were flushed and the water refilled. During the H_2_O_2_ exposure, water was aerated ensuring that oxygen levels remained above 7 mg L^−1^. After exposure, fish were monitored for 2 h for visual signs of stress *e.g.* flared opercula, increased ventilation, loss of equilibrium. Fish exposed to 1500 ppm H_2_O_2_ showed no change in behaviour, with fish exposed to 1700 and 1900 ppm exhibiting slightly increased ventilation. Rapid ventilation, loss of equilibrium and mortalities were observed following exposure to concentrations of 2100 ppm and above (total mortality for fish exposed to 2100–2700 ppm: diploids 25.0%; triploids 16.7%). As such, a nominal concentration of 1800 ppm was selected for the acute stress response trial.

Water samples were collected from each tank following the addition of H_2_O_2_ and the H_2_O_2_ concentration was immediately measured by cerium sulphate titration method [25]. To this end, 5 ml of 5 N H_2_SO_4_ and 7.5 ml of cerium IV sulphate solution were mixed in a conical flask. Then, a burette was filled with 50 ml of water sample and was slowly dispensed into the cerium IV sulphate solution, swirling to mix until the solution went colourless. The reading of the burette was then recorded and H_2_O_2_ concentration calculated.

#### 2.3.2 Acute stress response test

**Fig. 1:**
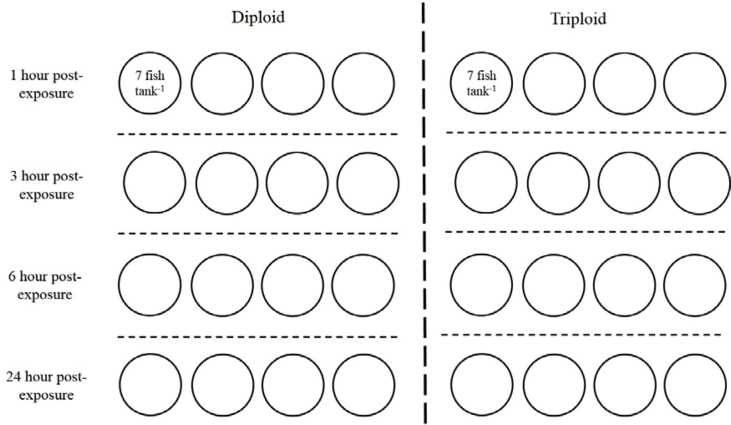
Schematic representation of experimental tank set-up for diploid and triploid Atlantic salmon showing 4 tanks allocated to each sampling time-point with 7 fish in each tank. Dotted lines used to differentiate tanks between sampling time-points (hours post exposure) and ploidy.

The acute stress response challenge was performed in August 2016 with ambient water temperature (14.0 °C) and simulated natural photoperiod set at 17 h light: 7 h darkness. For the H_2_O_2_ challenge test, 112 diploid (183.0 ± 5.6 g body weight and 266.7 ± 2.4 mm body length) and 112 triploid (215.0 ± 4.9 g body weight and 282.4 ± 2.2 mm body length) Atlantic salmon were used. Experimental fish were randomly allocated into 16 tanks (397 L cylindrical tanks, 1 m diameter, 0.4 m depth) per ploidy (n = 7 fish tank^−1^) to test the effects of H_2_O_2_ exposure in quadruplicate at four different time post-exposure. Following allocation, all fish were acclimated in trial tanks for 1 week prior to H_2_O_2_ exposure. On the day of H_2_O_2_ challenge, all fish were exposed to H_2_O_2_ at 09:00 h and then sampled at 1, 3, 6 and 24 h post-exposure (h.p.e) (n = 4 tanks time-point^−1^; 28 fish) (Fig. 1). Different tanks were sampled at each time-point to avoid stress induced by repeat netting of the fish. The water in the tanks was turned off and lowered to a set volume (200 L) before fish were exposed to a nominal concentration of 1800 ppm for 20 min after which the tanks were flushed and refilled with clean water. During the H_2_O_2_ exposure, water was aerated ensuring that oxygen level remained above 7 mg L^−1^. Water samples were collected from each tank following the addition of H_2_O_2_ and the concentration was immediately measured by cerium sulphate titration method [25], as previously described. The measured concentration across all experimental tanks was 1807 ± 83.9 ppm.

At each time-point, all 28 fish per ploidy were culled by lethal anaesthesia (MS-222, 1000 ppm, PHARMAQ, Norway) before being sampled. Fish from each tank (n = 4 tanks ploidy^−1^; 7 fish tank^−1^) (Fig. 1) were sampled within 15 min post-cull. Blood samples were obtained from the caudal vein using heparinised needles and syringes. An aliquot of blood was used for glucose and lactate measurements *in situ* while the remaining blood volume was kept on ice until centrifugation at 3000*g* for 10 min after which plasma was removed and frozen at −20 °C for cortisol analysis. Finally, small sections of the left second gill arch and liver were excised into RNAlater and kept on ice until further storage at −20 °C for gene expression analyses. On completion of the H_2_O_2_ challenge, the tanks were re-stocked with diploid and triploid Atlantic salmon (112 fish ploidy^−1^, 7 fish tank^−1^) (Fig. 1). All fish were acclimated for 1 week before the trial with ambient water temperature (14.0 °C) and simulated natural photoperiod set at 17 h light: 7 h darkness. All samplings were repeated in full for the diploid and triploid control groups. To this end, at 09:00 h on the sampling day, control fish were subjected to water volume reduction, aeration, water flushing and refilling but H_2_O_2_ was not added, and then fish were sampled 1, 3, 6 and 24 h later. At each time-point, 28 control diploid and triploid salmon were sampled as described previously (n = 4 tanks ploidy^−1^) (Fig. 1).

### 2.4 Glucose and lactate

Blood glucose and lactate were measured immediately after extraction by means of handheld meters: Contour^®^ USB (Bayer HealthCare, UK) and LactatePro™ 2 (Arkray Europe, The Netherlands), respectively. Firstly, an appropriate test strip was inserted into each device (Contour test strips and Lactate Pro 2 test strips, respectively). A small volume of whole blood (∼1 μl) was then extracted from the aliquot and applied to each test strips. After a few seconds, the measurement was displayed on each device screen and was recorded. New test strips were used for each fish. These had been previously validated for Atlantic salmon samples using enzymatic-colorimetric commercial test kits: Glucose (GO) Assay Kit (Sigma) and Lactate Dry-Fast (Sentinel Diagnostics, Italy) [25].

### 2.5 Cortisol

Plasma cortisol levels were measured with a commercial ELISA kit (RE52061, IBL-International, Hamburg, Germany). This kit had been previously used to quantify plasma cortisol in Atlantic salmon [46,47]. Following assay development, absorbance was measured at 450 nm using the Gen5 software and BioTek Synergy HT Microplate reader. The Gen5 Data Analysis Software was then used to calculate the concentration of cortisol in each sample based on the known standard curve concentrations, the dilutions made and the absorbances obtained. The intra- and inter-assay coefficients of variation were 2.6–3.5% and 2.1–5.0%, respectively (n = 20).

### 2.6 Lysozyme

Lysozyme activity in serum samples was measured turbidimetrically according to Morgan et al. [48]. Following completion of the assay, the reduction in absorbance at 540 nm was measured at 1 min intervals for 5 min using the Gen5 software and BioTek Synergy HT Microplate reader. One unit of lysozyme activity is defined as the amount of sample causing a decrease in absorbance at 0.001/min. Activity is expressed as units min^−1^ ml^−1^.

### 2.7 Gene expression

Liver and gill samples were homogenised in 1 ml of TriReagent (Sigma Aldrich, UK) and RNA was extracted in accordance with the manufacturer's instruction. RNA pellets were rehydrated in MilliQ water (250 μl liver samples; 75 μl gill samples) and incubated at 55 °C for 5 min then at room temperature for 40 min with gentle flicking of the tubes every 10 min to aid resuspension. Total RNA concentration was determined using a ND-1000 Nanodrop spectrophotometer (Labtech Int., East Sussex, UK) and RNA integrity (300 ng in 5 μl) was assessed by electrophoresis. To eliminate genomic DNA contamination, total RNA was then DNase treated (DNA-free™ kit, Thermo Fisher Scientific, Waltham, USA) following the manufacturer's instructions. The concentration of RNA was then assessed (ND-1000 Nanodrop spectrophotometer) to facilitate cDNA synthesis. The relative expression of 6 oxidative stress markers (*cat*, *gpx1*, *gr*, *hsp70, sod1* and *sod2*) and 4 immune genes (*saa5*, *crp/sap1a*, *crp/sap1b* and *il1β*) was determined in liver and gills from fish of all treatments, along with 5 reference genes: *β-actin*, *ef1a*, *rpl1*, *rpl2* and *b2m*. The primers used to amplify *cat*, *gpx1*, *gr*, *hsp70* were previously tested and validated for Atlantic salmon [25,[49], [50], [51]]. The primers used to amplify *sod1*, *sod2*, *saa5*, *crp/sap1a*, *crp/sap1b* and *il1β* were designed *de novo* using software PRIMER3 [52] and their target specificity was checked *in silico* using Blast (NCBI). cDNA was reverse transcribed from 1 μg of DNase-treated total RNA using random hexamer and Oligo (dT) 12–18 primers in a 20 μl total reaction volume (High-Capacity cDNA Reverse Transcription kit, Thermo Fisher Scientific, Waltham, USA). Real-time PCR was performed using Luminaris colour Higreen qPCR Master mix (Thermo Fisher Scientific, Waltham, USA). Reactions were run in duplicate in a LightCycler 480 thermocycler (Roche, UK) programmed to perform the following protocol: 50 °C for 2 min, 95 °C for 1 min, followed by 40 cycles at 95 °C for 15 s, annealing at X °C for 30 s (Table 1) and extension at 72 °C for 30 s. This was followed by a temperature ramp from 70 to 90 °C for melt-curve analysis to verify that no primer-dimer artefacts were present and only one product was generated from each qPCR assay. The final volume of the PCR reaction was 10 μL: 2.5 μl of cDNA, 5 μl of the qPCR Master Mix, 1.5 μl H_2_O and 0.5 μl each of forward and reverse primers (Table 1). Amplifications were carried out including systematic negative controls containing no cDNA (NTC, no template control). No primer-dimers occurred in the NTC. Gene expression quantification was achieved by including a parallel set of reactions containing serial dilutions from all pooled cDNA experimental samples and assigning each dilution the appropriate value of relative units (RUs). As a result, an estimated number of relative copies, corrected for the efficiency of the reaction, was automatically calculated for each sample. The normalized expression values were generated by the ΔCt method [53] and the results expressed as mean normalized ratios (±SE) between the RUs of target genes and a reference gene index calculated from the geometric mean of the most stable reference genes (*i.e*. *b2m*, *rpl2* and *rpl1* for diploid liver; *β-actin*, *rpl1* and *rpl2* for diploid gill; *ef1α* and *rpl2* for triploid liver; *ef1α* and *b2m* for triploid gill). Housekeeping genes stability was determined applying a correction for efficiency to the raw Ct standard deviation [54] using RefFinder [55]. Fold change differences between control and H_2_O_2_-exposed groups were also calculated at each time-point in both ploidy.

**Table 1:** Atlantic salmon primer sequences used for real-time PCR.

Gene	Name	Known function in vertebrates	Accession	F/R	Primer	Anneal (°C)
*β-actin*	*beta-actin*	Mediator of internal cell motility and growth	AF012125	F	ATCCTGACAGAGCGCGGTTACAGT	60
				R	TGCCCATCTCCTGCTCAAAGTCCA	
*ef1α*	*elongation factor 1 alpha*	Mediate recruitment of aminoacyl-tRNA to A-site of 80 S ribosome	DQ834870	F	CACCACCGGCCATCTGATCTACAA	60
				R	TCAGCAGCCTCCTTCTCGAACTTC	
*rpl1*	*RNA polymerase 1*	Transcription of ribosomal RNA	NM_001140826.1	F	ACTATGGCTGTCGAGAAGGTGCT	60
				R	TGTACTCGAACAGTCGTGGGTCA	
*rpl2*	*RNA polymerase 2*	Catalyses transcription of DNA to precursors of mRNA	BT049591.1	F	TAACGCCTGCCTCTTCACGTTGA	60
				R	ATGAGGGACCTTGTAGCCAGCAA	
*b2m*	*beta 2-microglobulin*	Cell surface protein; essential component for stable surface transport and expression	BT046451.2	F	TCCCAGACGCCAAGCAG	60
				R	TGTAGGTCTTCAGATTCTTCAGG	
*cat*	*catalase*	Catalyses decomposition of H_2_O_2_ to water and oxygen; protection of cells from oxidative damage	BT059457	F	CCCAAGTCTTCATCCAGAAACG	60
				R	CGTGGGCTCAGTGTTGTTGA	
*gpx1*	*glutathione peroxidase 1*	Protection of organism from oxidative damage; H_2_O_2_ detoxification	DW5665563	F	GCCCACCCCTTGTTTGTGTA	60
				R	AGACAGGGCTCCACATGATGA	
*gr*	*glutathione reductase*	Catalyses reduction of glutathione disulphide to the sulfhydryl form of glutathione, critical in resisting oxidative stress	XM014199133.1	F	CCAGTGATGGCTTTTTTGAACTT	60
				R	CCGGCCCCCACTATGA	
*hsp70*	*heat shock protein 70*	Important in protein folding and in protecting cells from stress	BG933934	F	CCCCTGTCCCTGGGTATTG	60
				R	CACCAGGCTGGTTGTCTGAGT	
*sod1*	*superoxide dismutase 1*	1^st^ line defence against reactive oxygen species (ROS); breakdown of potentially harmful ROS in cells	Q3ZLR1	F	GACCCCACTCTATCATCGGC	60
				R	AATAACTCCACAGGCCAGGC	
*sod2*	*superoxide dismutase 2*		C0H894	F	CTGGGCTTCGACAAGGAGAG	60
				R	GCTCACGTTCTCCCAGTTGA	
*saa5*	*serum amyloid A-5*	Acute phase protein; Secreted during acute phase of inflammation; integral part of innate immune response	B9EPA2	F	ACAAGTACTTCCACGCTCGG	60
				R	TCCTCATGTCCTCGACCACT	
*crp/sap1a*	*C-reactive protein (CRP)/serum amyloid P 1a*	Acute phase protein; Binds to molecules on dead/dying cells and activates the complement system	P79905	F	GGGAGCGTCACTGGATTTCA	60
				R	AGAATCCTCCGTGCACTTCG	
*crp/sap1b*	*C-reactive protein (CRP)/serum amyloid P* 1 b		B5X672	F	GTGGATGGAGAAGCTGCTGT	60
				R	GCTTGTCTCGACTGGGATGA	
*il1β*	*interleukin 1 beta*	Cytokine; important mediator of inflammatory response and involved in cell proliferation	Q6IWH5	F	TGAAGTCCATCAGCCAGCAG	60
				R	GGATGGTGAAGGTGGTGAGG	

### 2.8 Statistical analysis

Minitab software version 16 (Minitab Inc., Pennsylvania) was used in this study to perform basic descriptive statistics and comparisons using a significance level of 5% (*p* = 0.05). Prior to analysis, datasets were checked for normality using the Anderson-Darling test. Total mortality (%) was arcsine transformed for normality then non-parametric Kruskal-Wallis and Dunn's multiple comparison post-hoc test were used to assess ploidy and time differences. For cortisol, glucose, lactate and lysozyme activity, ANOVA manipulated by a GLM was carried out to analyse possible interactions between experimental groups and time-points. For this, ploidy, treatment (control or H_2_O_2_-exposed) and time (h.p.e) were considered fixed factors and tank considered as a random factor. Statistical differences in plasma cortisol levels, blood glucose and lactate levels and lysozyme activity between sampling time-points for a given experimental group were analysed by one-way ANOVA and Tukey post-hoc test. At each time-point, cortisol, glucose, lactate and lysozyme activity were also compared between experimental groups (diploid H_2_O_2_-exposed, diploid control, triploid H_2_O_2_-exposed, triploid control) by further one-way ANOVA. Statistical differences in gene expression levels between sampling time-points were analysed by one-way ANOVA. Additionally, for a given ploidy, gene expression levels were compared between treatments at each sampling time-point, using 2-sample t-tests. While the effects of polyploidy on gene expression have been well studied in plants, there is an overall lack of information regarding triploid gene expression in fish species. As such, it was not deemed appropriate to make direct comparisons between diploid and triploid gene expression in this study, and therefore the results will be presented individually.

## 3 Results

### 3.1 Cortisol

A significant effect of treatment and time on cortisol levels was observed throughout the trial. However, no significant effects of ploidy or the interaction between ploidy, treatment and time were observed.

**Fig. 2:**
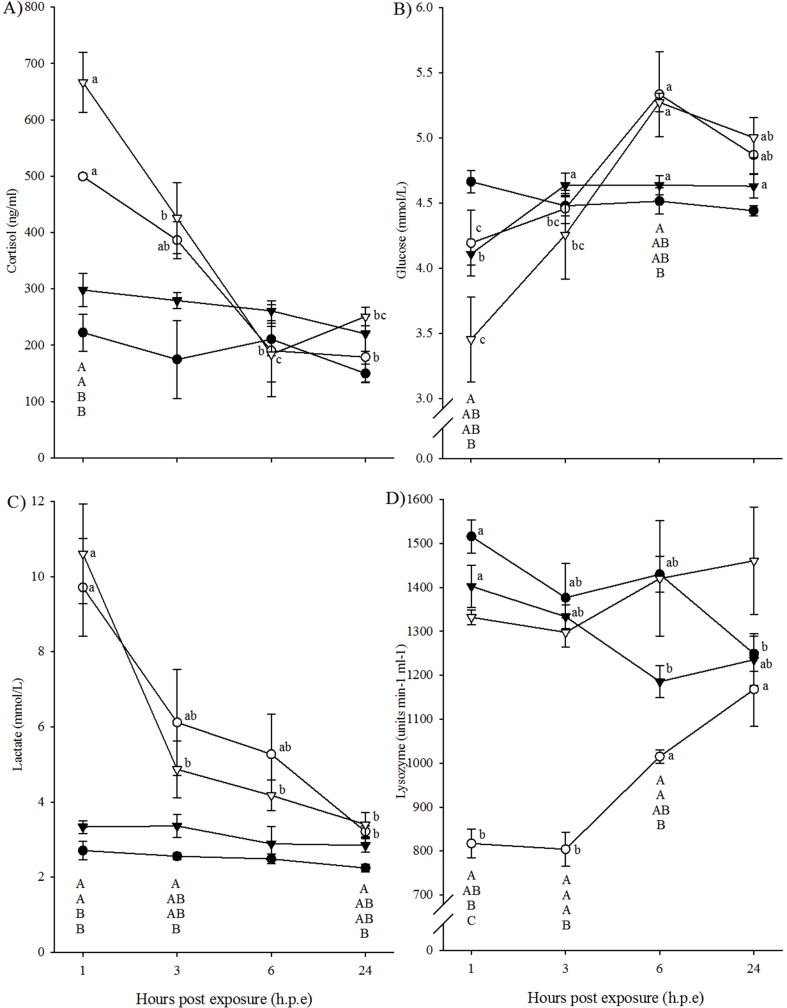
Plasma cortisol (A), blood glucose (B), blood lactate (C) levels and plasma lysozyme activity (D) (mean ± SEM, n = 4) over time in control (black) and H_2_O_2_-exposed (open), diploid (circle) and triploid (triangle) Atlantic salmon. Significant differences between experimental groups at a particular time-point are indicated by different capital letters, with the order of letters corresponding to the order of symbols within the time-point (one-way ANOVA *p* < 0.05). Significant differences between time-points (h.p.e) for an experimental group are indicated by different lowercase letters (one-way ANOVA, *p* < 0.05).

In diploids, plasma cortisol levels at 1 h. p.e were significantly higher (1.8 fold) in the H_2_O_2_-exposed fish (499.4 ± 5.3 ng/ml) than in the control fish (274.5 ± 43.9 ng/ml) (Fig. 2A). Within the diploid H_2_O_2_-exposed fish, significantly lower cortisol levels were observed at 6 h. p.e (190.0 ± 81.7 ng/ml) and 24 h. p.e (178.8 ± 44.4 ng/ml) compared to 1 h. p.e, while levels remained steady in the control fish.

In the triploids, similar patterns of cortisol to the diploids were observed. At 1 h. p.e, cortisol levels in the H_2_O_2_-exposed fish (666.4 ± 52.8 ng/ml) were significantly higher (2.2 fold) than in the control fish (297.9 ± 29.4 ng/ml) (Fig. 2A). Within the triploid H_2_O_2_-exposed fish, cortisol levels were significantly lower at 3 h. p.e (425.6 ± 63.3 ng/ml), 6 h. p.e (183.7 ± 49.1 ng/ml) and 24 h. p.e (250.8 ± 16.3 ng/ml) relative to 1 h. p.e, with cortisol levels at 6 h. p.e also significantly lower than at 3 h. p.e.

### 3.2 Glucose

A significant effect of time on blood glucose levels was observed throughout the trial. However, no significant effect of ploidy, treatment or the interaction between ploidy, treatment and time was found.

In diploids, blood glucose levels at 6 h. p.e were significantly higher in the H_2_O_2_-exposed fish (5.3 ± 0.3 mmol/L) than in the control fish (4.5 ± 0.1 mmol/L) (Fig. 2B). Within the diploid H_2_O_2_-exposed fish, glucose levels were significantly higher at 6 h. p.e compared to 1 h. p.e (4.2 ± 0.3 mmol/L) and 3 h. p.e (4.5 ± 0.1 mmol/L), with glucose levels at 24 h. p.e (4.9 ± 0.1 mmol/L) also significantly higher than at 1 h. p.e. Time did not significantly affect the diploid control group.

In triploids, no significant differences were found between H_2_O_2_-exposed and control groups throughout the study. Within the triploid H_2_O_2_-exposed fish, glucose levels at 1 h. p.e (3.5 ± 0.3 mmol/L) and 3 h. p.e (4.3 ± 0.3 mmol/L) were significantly lower than at 6 h. p.e (5.3 ± 0.1 mmol/L) (Fig. 2B), with levels at 1 h. p.e also significantly lower than at 24 h. p.e (5.0 ± 1.5 mmol/L). In the control fish, glucose levels were significantly higher at 3, 6 and 24 h. p.e (approximately 4.6 mmol/L) compared to 1 h. p.e (4.1 ± 0.1 mmol/L).

### 3.3 Lactate

A significant effect of treatment and time on blood lactate levels was evident throughout the trial. However, no significant effect of ploidy or the interaction between ploidy, treatment and time was found.

In diploids, blood lactate levels were significantly higher at 1 (3.6 fold) and 3 h. p.e (2.3 fold) in the H_2_O_2_-exposed group (1 h. p.e: 9.7 ± 1.3 mmol/L; 3 h. p.e: 6.1 ± 1.4 mmol/L) than in the control fish (1 h. p.e: 2.7 ± 0.2 mmol/L; 3 h. p.e: 2.6 ± 0.1 mmol/L) (Fig. 2C). In addition, time had a significant effect on the diploid H_2_O_2_-exposed group with lactate level at 1 h. p.e significantly higher than at 24 h. p.e (3.2 ± 0.2 mmol/L), while time did not significantly affect lactate in the control group.

In triploids, a similar trend in blood lactate levels was observed. At 1 h. p.e, blood lactate levels in the H_2_O_2_-exposed fish (10.6 ± 1.3 mmol/L) were significantly higher (>3 fold) than the control fish (3.3 ± 0.2 mmol/L) (Fig. 2C). Within the triploid H_2_O_2_-exposed fish, blood lactate levels were significantly higher at 1 h. p.e than at the other three time-points (3.4–4.9 mmol/L), while time post-exposure did not significantly affect lactate levels in the control fish.

### 3.4 Lysozyme

Significant effects of ploidy and treatment on plasma lysozyme activity were evident throughout the trial (Fig. 2D). However, no significant effect of time or the interaction between ploidy, treatment and time was found.

Ploidy had a significant effect on lysozyme activity in the H_2_O_2_-exposed groups, with diploids exhibiting lower lysozyme at 1, 3 and 6 h. p.e (804–1015 units min^−1^ ml^−1^) compared to triploids (1298–1420 units min^−1^ ml^−1^) (Fig. 2D).

In diploids, lysozyme activity recorded for the H_2_O_2_-exposed fish at 1 h. p.e (817.1 ± 32.9 units min^−1^ ml^−1^), 3 h. p.e (804.3 ± 38.9 units min^−1^ ml^−1^) and 6 h. p.e (1015 ± 15.4 units min^−1^ ml^−1^) was significantly lower than the control fish at the same time-points (1376–1516 units min^−1^ ml^−1^) (Fig. 2D). Time also affected both diploid groups, with the H_2_O_2_-exposed fish showing significantly lower lysozyme activity at 1 and 3 h. p.e compared to at 6 and 24 h. p.e (1167.9 ± 83.9 units min^−1^ ml^−1^). The control fish showed significantly lower lysozyme at 24 h. p.e (1248.9 ± 39.6 units min^−1^ ml^−1^) than at 1 h. p.e (1515.7 ± 38.1 units min^−1^ ml^−1^).

In the triploid fish, lysozyme in the control fish was significantly lower at 6 h. p.e (1185.6 ± 36.3 units min^−1^ ml^−1^) and 24 h. p.e (1235.4 ± 58.8 units min^−1^ ml^−1^) than at 1 h. p.e (1402.5 ± 47.9 units min^−1^ ml^−1^), with no significant effect of time observed in the H_2_O_2_-exposed fish (Fig. 2D). Comparable lysozyme activity was recorded for the triploid H_2_O_2_-exposed and control groups at each time-point.

### 3.5 Mortality

Post-exposure to H_2_O_2_, mortalities occurred in all tanks in the diploid and triploid H_2_O_2_-exposed groups (Table 2), while no mortalities occurred in the diploid and triploid control groups. Statistical analysis found no significant effect of ploidy or time-point tank on total mortality (%).

**Table 2:** Total mortality (%) in the time-point tanks (n = 4, total of 28 fish) allocated to diploid and triploid H_2_O_2_-exposed groups (mean ± SEM).

Time-point tanks	Diploid	Triploid
1 h.p.e	3.6 ± 0.5	6.3 ± 1.3
3 h.p.e	13.4 ± 0.7	8.9 ± 1.3
6 h.p.e	10.7 ± 0.5	8.0 ± 0.7
24 h.p.e	7.1 ± 0.9	12.5 ± 1.1

### 3.6 Gene expression

#### 3.6.1 Diploid liver

**Fig. 3:**
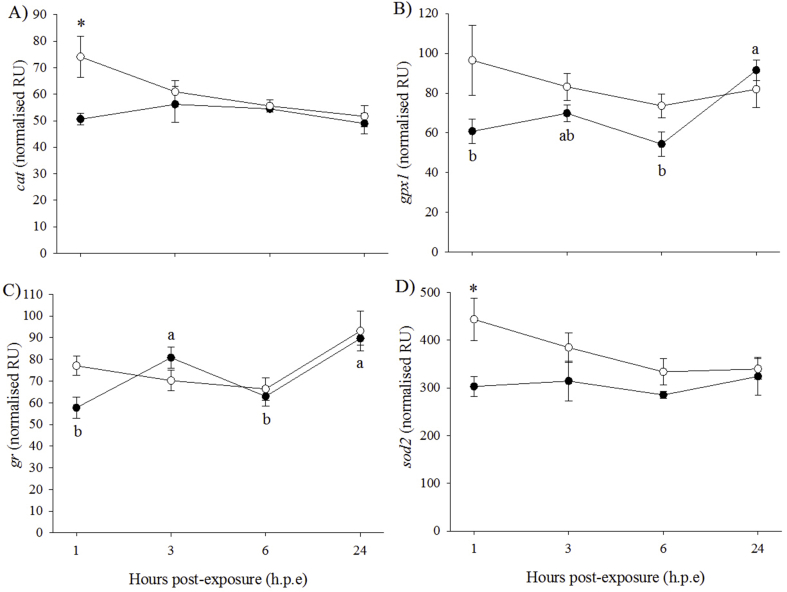
Gene expression (mean ± SEM, n = 4) of oxidative stress markers in liver of control (black circle) and H_2_O_2_-exposed (open circle) diploid salmon. Asterisks indicate significant differences between experimental groups at a particular time-point (two-sample *t*-test, *p* < 0.05). Superscript letters indicate significant differences between time-points (h.p.e) within the control group (one-way ANOVA, *p* < 0.05).

The expression of *cat* was significantly higher at 1 h. p.e in the H_2_O_2_-exposed fish (74.1 ± 7.6 normalised relative unit (NRU)) compared to the control fish (50.6 ± 2.1 NRU) (Fig. 3A). However, no significant differences in *cat* expression levels were detected between time-points in either group. Expression of *gpx1* in the control fish at 24 h. p.e (91.5 ± 5.1 NRU) was significantly higher than at 1 h. p.e (60.8 ± 6.1 NRU) and 6 h. p.e (54.4 ± 6.1 NRU) (Fig. 3B). Expression of *gr* in the control fish at 3 h. p.e (80.8 ± 4.8 NRU) and 24 h. p.e (89.7 ± 3.1 NRU) was significantly higher than at 1 h. p.e (57.7 ± 4.9 NRU) and 6 h. p.e (62.9 ± 4.5 NRU) (Fig. 3C). No significant differences in the expression of *gpx1* and *gr* were detected between the H_2_O_2_-exposed and control groups. The expression of *sod2* in the H_2_O_2_-exposed fish (443.8 ± 44.8 NRU) at 1 h. p.e was significantly higher than in the control fish (303.2 ± 21.5 NRU) (Fig. 3D). No significant differences in *hsp70* and *sod1* expression were found between treatment groups or time-points (data not shown).

For the immune genes investigated (*saa5*, *crp/sap1a* and *crp/sap1b*), variable patterns of expression were exhibited and no significant effects were observed for any of these genes analysed in the liver of diploid fish (data not shown).

#### 3.6.2 Diploid gill

**Fig. 4:**
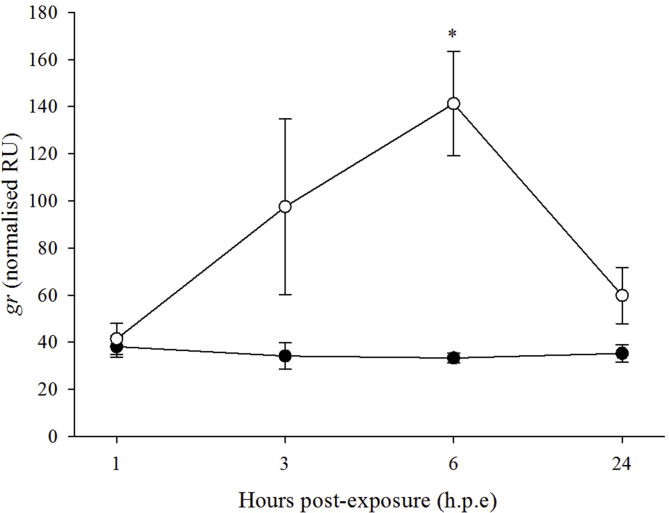
Gene expression (mean ± SEM, n = 4) of an oxidative stress marker in gill of control (black circle) and H_2_O_2_-exposed (open circle) diploid salmon. Asterisks indicate significant differences between experimental groups at a particular time-point (two-sample *t*-test, *p* < 0.05).

The expression of g*r* was significantly higher in the H_2_O_2_-exposed fish (141.3 ± 22.0 NRU) than in the control fish (33.3 ± 1.9 NRU) at 6 h. p.e (Fig. 4). Time did not have a significant effect on *gr* expression in both the H_2_O_2_-exposed and control fish. Fold changes (FC) differences in *gr* expression for the H_2_O_2_-exposed group relative to the control fish were 3.1, 5.1 and 2.0 at 3, 6 and 24 h. p.e, respectively. No significant effects of treatment or time were observed for any of the other oxidative stress markers (*cat*, *gpx1*, *hsp70*, *sod1* and *sod2*).

**Fig. 5:**
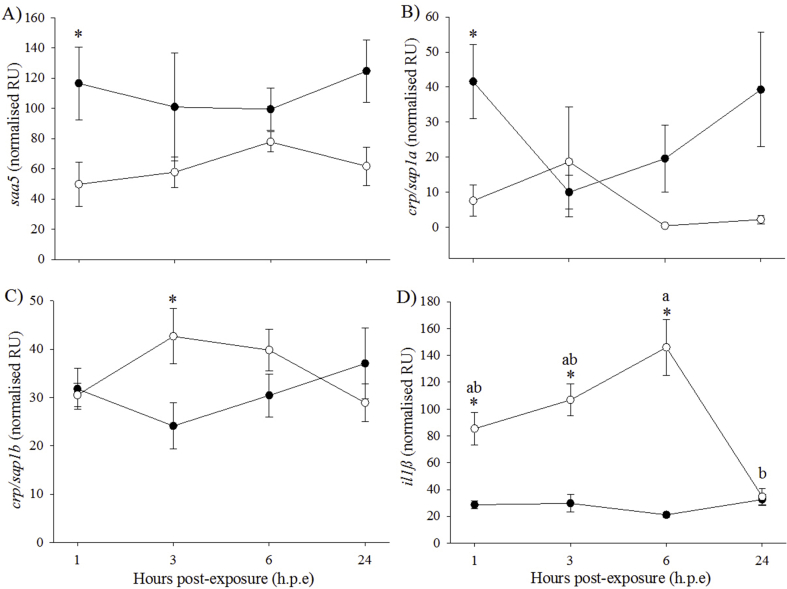
Gene expression (mean ± SEM, n = 4) of immune markers in gill of control (black circle) and H_2_O_2_-exposed (open circle) diploid salmon. Asterisks indicate significant differences between experimental groups at a particular time-point (two-sample *t*-test, *p* < 0.05). Superscript letters indicate significant differences between time-points (h.p.e) within the H_2_O_2_-exposed group (one-way ANOVA, *p* < 0.05).

As for immune genes, significantly higher expression of *saa5* was observed in the control fish than H_2_O_2_-exposed fish at 1 h. p.e (control: 116.5 ± 24.1 NRU; H_2_O_2_-exposed: 49.8 ± 14.6 NRU) and 24 h. p.e (control: 124.8 ± 20.7 NRU; H_2_O_2_-exposed: 61.8 ± 12.7 NRU) (Fig. 5A). The expression of *crp/sap1a* was also significantly higher in the control group (41.6 ± 10.7 NRU) than in the H_2_O_2_-exposed group (7.5 ± 4.5 NRU) at 1 h. p.e (Fig. 5B). The expression of c*rp/sap1b* at 3 h. p.e was significantly higher in H_2_O_2_-exposed fish (42.7 ± 5.7 NRU) than control fish (24.1 ± 4.7 NRU) (Fig. 5C). No significant differences between time-points were found for *saa5*, *crp/sap1a* and *crp/sap1b* expression. For *il1β*, expression was significantly higher in the H_2_O_2_-exposed fish than the control fish at 1 h. p.e (H_2_O_2_-exposed: 85.4 ± 12.2 NRU; control: 28.6 ± 2.7 NRU), 3 h. p.e (H_2_O_2_-exposed: 106.8 ± 11.9 NRU; control: 29.7 ± 6.4 NRU) and 6 h. p.e (H_2_O_2_-exposed: 146 ± 20.8 NRU; control: 21.1 ± 1.8 NRU) (Fig. 5D). Significant differences between time-points were detected in the H_2_O_2_-exposed fish, with expression of *il1β* significantly lower at 24 h. p.e (34.6 ± 6.4 NRU) than at 6 h. p.e. FC in *il1β* expression for the H_2_O_2_-exposed/control group were 2.7, 4.2 and 8.2 at 1, 3 and 6 h. p.e, respectively.

#### 3.6.3 Triploid liver

**Fig. 6:**
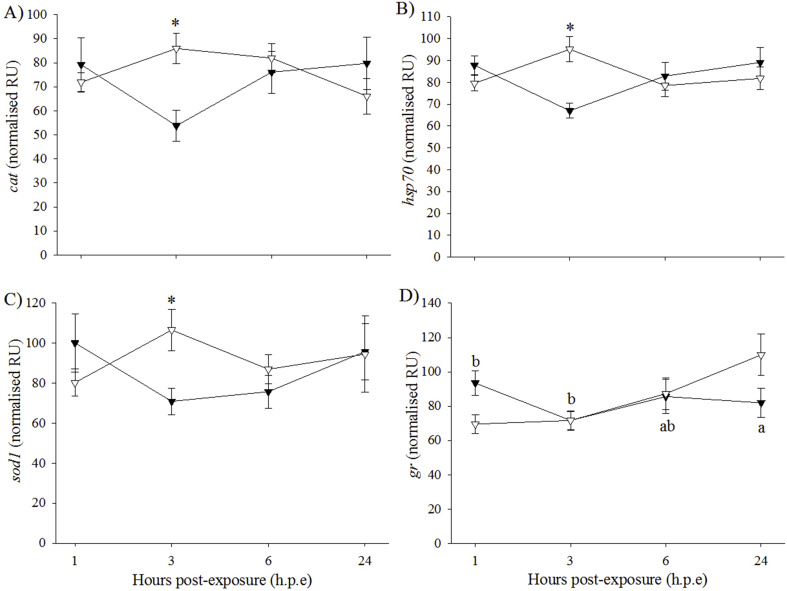
Gene expression (mean ± SEM, n = 4) of oxidative stress markers in liver of control (black triangle) and H_2_O_2_-exposed (open triangle) triploid salmon. Asterisks indicate significant differences between experimental groups at a particular time-point (two-sample *t*-test, *p* < 0.05). Superscript letters indicate significant differences between time-points (h.p.e) within the control group (one-way ANOVA, *p* < 0.05).

At 3 h. p.e, the expression levels of *cat*, *hsp70* and *sod1* were significantly higher in the H_2_O_2_-exposed fish (*cat*: 85.9 *±* 6.3 NRU; *hsp70*: 95.1 *±* 5.7 NRU; *sod1*: 106.5 *±* 10.3 NRU) than in the control fish (*cat*: 53.8 *±* 6.4 NRU; *hsp70*: 67.0 *±* 3.3 NRU; *sod1*: 70.9 *±* 6.6 NRU) (Fig. 6A, B, C). For these three genes, no significant differences were found between time-points for either experimental group. The expression of *gr* in the control fish at 24 h. p.e (81.9 *±* 8.5 NRU) was significantly different to the expression recorded at 1 h. p.e (93.5 *±* 7.3 NRU) and 3 h. p.e (71.7 *±* 5.7 NRU) (Fig. 6D). There were no significant effects of treatment or time on *gpx1* or *sod2* (data not shown).

**Fig. 7:**
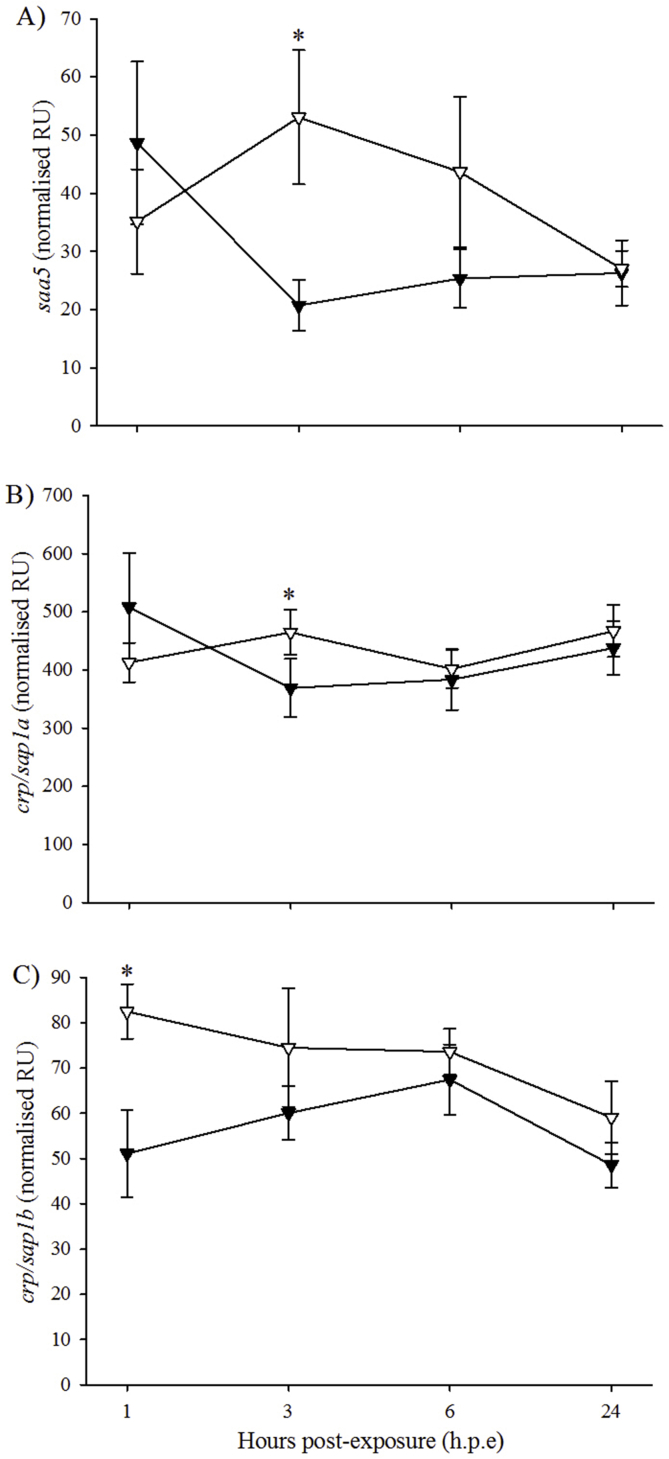
Gene expression (mean ± SEM, n = 4) of immune markers in liver of control (black triangle) and H_2_O_2_-exposed (open triangle) triploid salmon. Asterisks indicate significant differences between experimental groups at a particular time-point (two-sample *t*-test, *p* < 0.05).

At 3 h. p.e, the expression of *saa5* was significantly higher in the H_2_O_2_-exposed fish (53.1 *±* 11.5 NRU) than in the control fish (20.8 *±* 4.4 NRU) (Fig. 7A). This difference was also observed in the expression of *crp/sap1a*, with expression levels in the H_2_O_2_-exposed fish at 3 h. p.e (464.9 *±* 38.6 NRU) significantly higher than the control fish (369.1 *±* 49.8 NRU) (Fig. 7B). At 1 h. p.e, the expression of *crp/sap1b* was significantly higher in the H_2_O_2_-exposed fish (82.5 *±* 6.0 NRU) than in the control fish (51.1 *±* 9.6 NRU) (Fig. 7C).

#### 3.6.4 Triploid gill

**Fig. 8:**
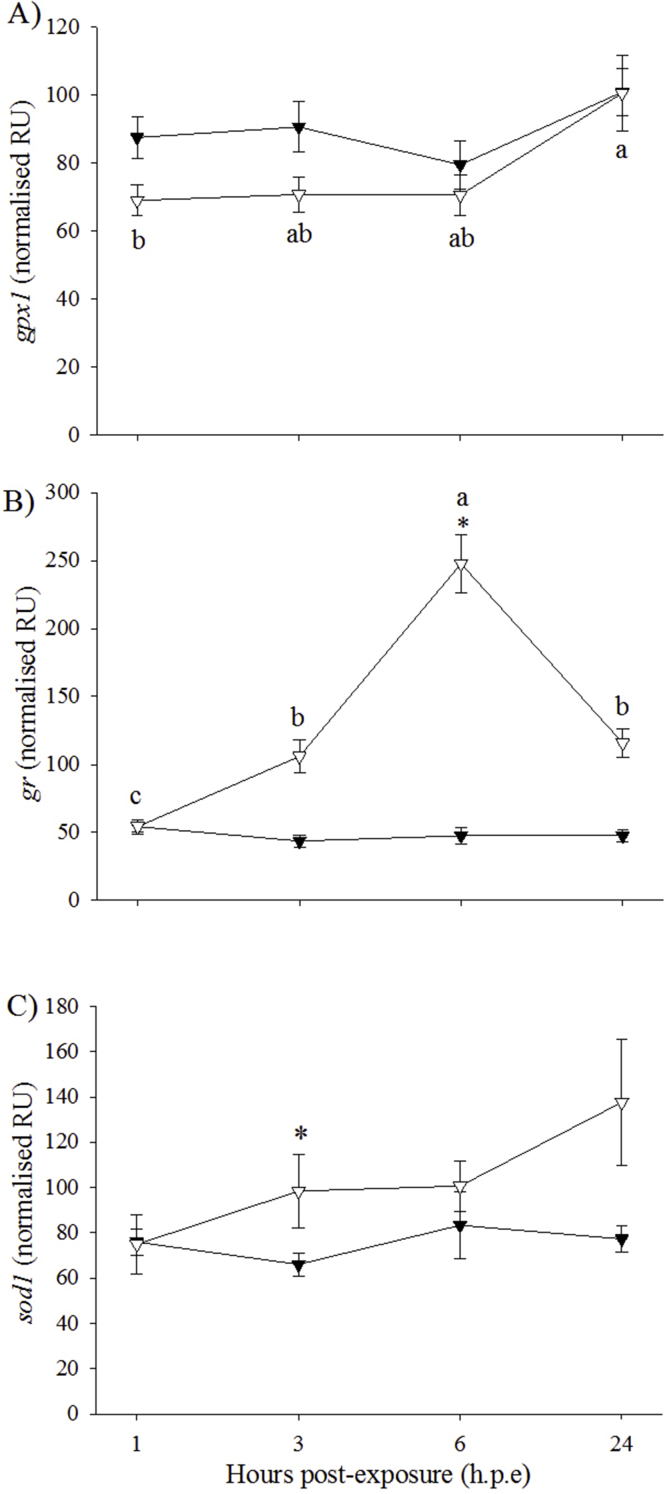
Gene expression (mean ± SEM, n = 4) of oxidative stress markers in gill of control (black triangle) and H_2_O_2_-exposed (open triangle) triploid salmon. Asterisks indicate significant differences between experimental groups at a particular time-point (two-sample *t*-test, *p* < 0.05). Superscript letters indicate significant differences between time-points (h.p.e) within the H_2_O_2_-exposed group (one-way ANOVA, *p* < 0.05).

The expression of *gpx1* in the control fish was significantly lower at 1 h. p.e (87.6 *±* 6.2 NRU) than at 24 h. p.e (100.9 *±* 7.0 NRU) (Fig. 8A). In addition, the expression of *gpx1* at 1 h. p.e was significantly higher in the control group than in the H_2_O_2_-exposed group (69.1 *±* 4.6 NRU). The expression of *gr* was significantly higher in the H_2_O_2_-exposed group than the control group at 3, 6 and 24 h. p.e (H_2_O_2_-exposed average: 156.5 ± 14.7 NRU; control average: 46.2 ± 4.9 NRU) (Fig. 8B). Within the H_2_O_2_-exposed group, *gr* expression was significantly higher at 3 h. p.e and 24 h. p.e compared to 1 h. p.e (54.3 ± 3.7 NRU), with expression at 6 h. p.e significantly higher than at the other time-points. FC in *gr* expression for the H_2_O_2_-exposed/control group were 2.1, 5.9 and 4.4 at 3, 6 and 24 h. p.e, respectively. Regarding *sod1*, expression level at 3 h. p.e was significantly higher in the H_2_O_2_-exposed group (98.4 ± 16.3 NRU) than in the control group (65.9 ± 5.1 NRU) (Fig. 8C). There were no significant effects of treatment or time on the expression of the other oxidative stress markers (*cat*, *hsp70* and *sod2*) (data not shown).

**Fig. 9:**
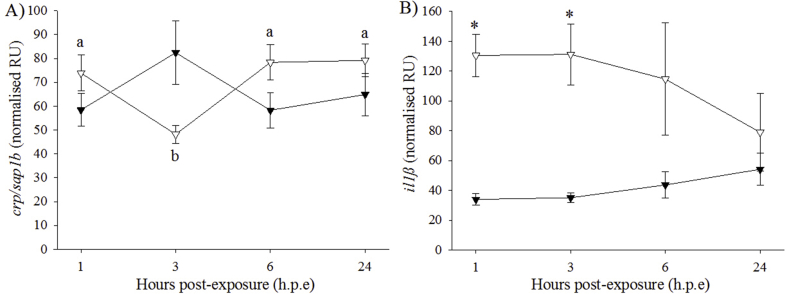
Gene expression (mean ± SEM, n = 4) of immune markers in liver of control (black triangle) and H_2_O_2_-exposed (open triangle) triploid salmon. Asterisks indicate significant differences between experimental groups at a particular time-point (two-sample *t*-test, *p* < 0.05). Superscript letters indicate significant differences between time-points (h.p.e) within the H_2_O_2_-exposed group (one-way ANOVA, *p* < 0.05).

The expression of *crp/sap1b* in the H_2_O_2_-exposed group was significantly lower at 3 h. p.e (48.2 ± 3.8 NRU) than at 1, 6 and 24 h. p.e (74.0–79.0 NRU) (Fig. 9A). At 1 h. p.e and 3 h. p.e, the expression of *il1β* was significantly higher in the H_2_O_2_-exposed group (1 h. p.e: 130.6 *±* 14.1 NRU; 3 h. p.e: 131.3 *±* 20.1 NRU) than in the control group (1 h. p.e: 34.1 *±* 3.8 NRU; 3 h. p.e: 35.1 *±* 3.2 NRU) (Fig. 9B). FC in *il1β* expression for the H_2_O_2_-exposed/control group were 3.9, 3.8, 3.3 and 1.2 at 1, 3, 6 and 24 h. p.e, respectively. There were no significant effects of treatment or time on the expression of the other immune markers (*saa5* and *crp/sap1a*) (data not shown).

## 4 Discussion

This study compared the stress and immune responses of diploid and triploid Atlantic salmon following experimental exposure to H_2_O_2_. The primary (cortisol) and secondary (glucose and lactate) stress indicators were not significantly influenced by ploidy while they were significantly impacted by time post-exposure. Lysozyme activity was significantly affected by ploidy and time post-exposure, although activity levels were within a normal range for Atlantic salmon. Hydrogen peroxide exposure significantly affected the expression of several stress and immune genes in the liver and gills of both diploid and triploid Atlantic salmon including catalase (*cat*), glutathione reductase (*gr*) and interleukin 1-beta (*il1β*).

Cortisol is the principal corticosteroid produced by fish and is thought to have numerous roles in the stress response pathway including energy mobilisation, stimulation of ion regulatory processes and facilitation of oxygen uptake [[56], [57], [58], [59]]. As a primary stress biomarker, cortisol is one of the most commonly used indicators of stress in teleost fish [59,60]. In this study, cortisol was significantly increased in the H_2_O_2_-exposed groups compared to the control groups at 1 h. p.e for both ploidy, before gradually decreasing over time to reach basal levels comparable to those observed in the control groups. This is consistent with numerous studies which have found elevated levels of cortisol in response to disease treatment with H_2_O_2_ [25,26,61] or other chemotherapeutants [62,63]. Ploidy did not have a significant effect on cortisol response and this is in accordance with previous studies investigating the primary stress response of triploid fish which reported similarly elevated cortisol in both diploid and triploid salmonids in response to handling [64], confinement [[64], [65], [66]], anaesthesia [67], transport [68] and sea water transfer [69].

Two well-studied secondary biomarkers of stress are glucose, an essential carbohydrate involved in the bioenergetics of animals which can be transformed into chemical (ATP) and mechanical energy [56,60,70] and lactate, the product of anaerobic metabolism, produced from pyruvate via the enzyme lactate dehydrogenase [65,70]. Similar glucose levels were observed in all groups until 6 h. p.e when levels in the H_2_O_2_-exposed groups increased above the controls, with a significant difference observed between the diploid H_2_O_2_-exposed and control groups, before returning to similar levels as the control groups at 24 h. p.e. The difference between exposed and control groups supports previous studies assessing the effects of H_2_O_2_ [25,26,61]. However, it should be noted that the response of glucose appeared to be delayed. It has previously been stated that secondary stress responses, such as glucose, happen over a slower timescale than primary stress responses [60] and the slow response in this study supports previous findings by Bowers et al. [26]. In addition, ploidy did not have a significant effect on glucose in this study, as observed in studies assessing physical and chemical stressors on the stress response of triploid salmonids [64,[66], [67], [68], [69]]. This finding is suggestive of similar stress-induced mobilisation of energy reserves between ploidy [64]. Lactate levels were significantly higher in the diploid and triploid H_2_O_2_-exposed fish compared to the control groups at 1 h. p.e, although lactate in the H_2_O_2_-exposed groups returned to basal levels by 24 h. p.e. This finding is supported by other studies assessing the effects of H_2_O_2_ [25,61] and other chemicals, such as metomidate, Aqui-S™ and clove oil [71], on the fish stress response. Despite the previous statement that secondary responses can occur over a slower timescale than primary responses [60], the pattern of lactate observed in this study appears to be more similar to the cortisol response than to the glucose profile, with a peak at 1 h. p.e rather than 6 h. p.e. As increases in lactate are known to occur in response to both exercise and stress [72], it could be suggested that the peak in lactate in this study was induced directly by the stress of H_2_O_2_ exposure as well as potentially increased swimming activity as a result of H_2_O_2_ exposure [73]. Furthermore, it is recognised that lactate production acts as a pathway for glyconeogenesis, a metabolic pathway producing glucose, therefore, the later peak in glucose may also be related to this mechanism [25,59,72]. Additionally, lactate was not significantly affected by ploidy in this study and this supports studies assessing the stress response of diploid and triploid Atlantic salmon following confinement [65,66] and the effects of exhaustive exercise in diploid and triploid rainbow trout [44]. Overall, the findings for these three stress biomarkers (cortisol, glucose and lactate) supports the ability of triploids to cope similarly to diploids in response to stress induced by H_2_O_2_ exposure.

While lysozyme, a bacteriolytic enzyme, is mainly considered as a component of the innate immune response, it is also considered as an indicator of stress [74]. However, it is recognised that lysozyme activity levels can be highly variable depending on intensity, duration and type of stress, and enhanced or suppressed lysozyme activity has been reported in the literature [69,[74], [75], [76], [77]]. In this study, variable patterns of lysozyme activity were recorded with significant effects of treatment and ploidy observed. The lysozyme activity recorded in the diploid H_2_O_2_-exposed group was significantly lower than their respective control group at 1, 3 and 6 h. p.e. This is in agreement with previous work by Yildiz [78] who observed decreased lysozyme levels in groups exposed to Leteux-Meyer mixture (formalin and malachite green) compared to the control groups. Significant ploidy effects were observed in the H_2_O_2_-exposed groups at 1, 3 and 6 h. p.e with diploids showing significantly lower activity than triploids. This concurs with a study by Taylor et al. [69] which found reduced lysozyme in diploid Atlantic salmon compared to triploids following seawater transfer stress.

It is recognised that, while H_2_O_2_ is an effective treatment for sea lice and AGD, it can become toxic and even lethal at water temperatures above 13.5 °C [19,20,79]. Despite this knowledge, given that water temperatures experienced by Atlantic salmon often exceed 13.5 °C in summer months in many salmon farming regions, and that sea lice infections occur more quickly at higher temperatures as well as the knowledge that sea lice are developing resistance against H_2_O_2_, it is recognised that treatments are performed at unsuitable temperatures and at higher doses than those prescribed [18,[80], [81], [82]]. Considering this, while water temperature in the current study (14 °C) was above the recommended temperature for exposure (14 °C), this study was undertaken to give a perspective of what could occur during a “normal” salmon production cycle. Mortalities occurred in both diploid and triploid Atlantic salmon following H_2_O_2_ exposure. This concurs with Bruno and Raynard [83] whose study found 35% mortality following H_2_O_2_ exposure at 13.5 °C, thus supporting the potentially lethal nature of H_2_O_2_ at high temperatures. However, while the mortalities may be linked to temperature, as no mortalities occurred in the dose-response toxicity test at 14 °C it is recognised that other factors may have contributed, including inter-fish differences in H_2_O_2_ tolerance and potentially compromised gill function [20]. The mortalities in this study highlight that the salmon farming industry must be cautious when treating with H_2_O_2_, particularly at high temperatures and doses. In addition, with triploid salmon known to be more sensitive to higher temperatures [45,84], it emphasises the need for studies assessing the combined effects of chemical treatments and varying temperatures in order to develop triploid specific farming protocols and determine the suitability of production sites, in terms of environmental profiles, to farm triploids.

It is recognised that exposure to H_2_O_2_ can impact the expression of oxidative stress genes in fish [25]. However, there is an overall lack of information regarding the effects of triploidy on gene expression in vertebrates [85,86]. While a few studies suggested the occurrence of a dosage effect on triploid gene expression [[85], [86], [87], [88], [89], [90]], the mechanisms underlying such dosage effects have not yet been elucidated. As such, it was not considered appropriate to directly compare diploid and triploid gene expression in this study, and so ploidy and tissue were assessed separately. In the liver of diploid salmon, there was a general trend for the H_2_O_2_-exposed group to show higher gene expression of oxidative stress markers at 1 h. p.e, significantly so in *cat* and *sod2*, before returning to levels similar to that of the control group. This is supported by a previous study which found elevated expression of oxidative stress genes in Atlantic salmon exposed to H_2_O_2_ compared to controls [25]. However, there was no significant changes in the expression patterns of selected immune genes assessed in the liver which is in contrast with a previous study reporting that acute phase proteins synthesised in the liver, such as *saa5*, *crp/sap1a* and *crp/sap1b*, are involved in the stress response [91]. Studies in carp (*Cyprinus carpio*), however, have shown that increases in cortisol can have a suppressive effect of the expression of acute phase proteins [92,93] and so it could be suggested that this may have occurred in diploid liver in the present study. In diploid gills, *gr* showed significantly higher expression in the H_2_O_2_-exposed fish than in the controls at 6 h. p.e which agrees with Tort et al. [94] who found increased glutathione in the gill of walleye (*Sander vitreus*) following exposure to H_2_O_2_. However, no significant effect of H_2_O_2_ was found in the other oxidative stress genes investigated. This finding contrasts with Vera and Migaud [25] whose study found elevated expression of a range of oxidative stress genes in the gills of Atlantic salmon. It could be suggested that the differences between the current study and Vera and Migaud [25] may be linked to the different fish populations (*e.g.* 110.3 ± 0.5 g in Vera and Migaud [25] and average 199 g in the current study) used as well as differences in experimental design (H_2_O_2_ exposures at set times throughout the day with sampling immediately afterwards) or temperature (12.3 ± 0.3 °C) but further studies would be required to confirm this. Regarding the immune genes, *saa5* and *crp/sap1* showed higher expression levels in the control group whereas the H_2_O_2_-exposed group showed higher expression levels of *crp/sap1b* and *il1β*. The enhanced expression reported for *il1β* is in agreement with previous studies which found increased *il1β* expression following vaccination and short-term handling stress [95,96]. This finding was also in agreement with a study investigating the role of *il1β* in acute stress in carp [97]. The authors suggest that increased expression of *il1β* may influence the activity of the hypothalamus–pituitary–interrenal axis, which is activated during stress and, in turn, potentially alter the release and production of cortisol [97].

For oxidative stress genes in triploid liver, the H_2_O_2_-exposed group showed higher expression of *cat*, *hsp70* and *sod1* than the control group at 3 h. p.e. This finding concurs with a previous study which found increased gene expression in H_2_O_2_-exposed diploid Atlantic salmon compared to controls [25]. However, it should be noted that this contrasts with diploids in the current study, where significant differences between treatment groups occurred at 1 h. p.e. This could have implications for triploid recovery post-exposure to H_2_O_2_. For example, the expression of superoxide dismutase 1 or 2 (*sod1*, *sod2*) and catalase (*cat*) were elevated in the livers of H_2_O_2_-exposed diploids at 1 h. p.e and triploids at 3 h. p.e. Following a stress event, superoxide dismutase converts the potentially damaging superoxide anion into oxygen and H_2_O_2_ and catalase then breaks down H_2_O_2_ into oxygen and water [98,99]. The delay in these enzymes observed in triploids may cause cells to be exposed to harmful levels of reactive oxygen species for extended periods of time which could result in cell injury or death, and DNA damage [100,101]. A similar delayed response in triploids was also detected in the hypoferraemic response, a bacterial defence mechanism [102]. In terms of the immune genes, the H_2_O_2_-exposed group showed significantly higher expression of *saa5* and *crp/sap1a* at 3 h. p.e and *crp/sap1b* at 1 h. p.e than the control group. While this finding appears to refute the suggestion of suppressive action by cortisol on acute phase proteins, it could be suggested that the acute phase response is more sensitive in triploids than in diploids. However, this suggestion would require further study to fully determine differences in the action of the acute phase response between diploid and triploid Atlantic salmon. In triploid gill, the expression of *sod1* and *gr* were significantly higher in the H_2_O_2_-exposed group compared to the controls at 3 and 6 h. p.e, respectively. This is supported by the finding from Tort et al. [94] who reported increased glutathione in the gill of walleye following H_2_O_2_ exposure. In terms of ploidy effects on FC in gene expression, results suggested a similar response of both diploid and triploid Atlantic salmon to H2O2 exposure. Thus, for *gr* expression, both ploidy showed similar FC at each time-point post-exposure. It could be suggested that the extra genetic material present in triploid cells is compensated for, so that gene expression becomes equal to diploids [87] but further studies are still required to fully elucidate gene expression in triploid salmon. No significant effect of H_2_O_2_ was found for the other oxidative stress genes investigated (*cat*, *gpx1*, *hsp70* and *sod2*), which is again in contrast to Vera and Migaud [25] who found increased expression of oxidative stress genes in the gills of diploid Atlantic salmon exposed to H_2_O_2_. As with diploids, *il1β* in the gills was the most reactive gene in response to H_2_O_2_ exposure, while the remaining three immune genes showed little changes. In terms of ploidy, FC in *il1β* expression were similar in diploid and triploid Atlantic salmon at 1 and 3 h. p.e, with higher FC in diploids at 6 h. p.e. However, the reason for this finding is unclear at this time and would require further research into the effect of triploidy on gene expression.

## 5 Conclusions

This study, representing the first testing of H_2_O_2_ in triploid Atlantic salmon, confirmed that exposure to H_2_O_2_ triggered primary and secondary stress responses (cortisol, glucose and lactate), and that these responses were not significantly influenced by ploidy. This suggests that the physiological response of triploids to cope with the stress induced by H_2_O_2_ exposure would be comparable to that observed in their diploid counterparts. This study also represents the first assessment of the effects of H_2_O_2_ exposure on the expression of oxidative stress and immune genes in triploids. While it was not considered appropriate to directly compare diploid and triploid gene expression, a difference was observed in the time response of certain genes between diploids and triploids, with triploids showing delayed increases in gene expression. This could suggest that triploids need longer to cope with the stress associated with H_2_O_2_ exposure but could also result in triploid cells being exposed to harmful levels of reactive oxygen species for extended periods which could cause cell and DNA damage as well as cell death. As such, studies are required to further assess the effect of triploidy on gene expression and to determine if other processes are delayed and the impact this may have on stress and disease resistance. Finally, as fish in this study were not infected by any pathogen, such as sea lice or AGD during the H_2_O_2_ exposure, as would normally be the case in commercial salmon farming operations, it is also recommended that studies be undertaken to assess the additive effect of pathogen challenge with treatment on both the immune and stress responses. Undertaking this type of research would aid in determining the ability of triploids to cope with combined stressors and, thus assess their overall robustness for commercial aquaculture production.

## Appendix A Supplementary data

Supplement1 - Efficiency table Supplement1 - Efficiency table

## References

[1] Bostock J., McAndrew B., Richards R., Jauncey K., Telfer T., Lorenzen K. (2010). Aquaculture: global status and trends, Philos. Trans. R. Soc. Lond. B. Biol. Sci..

[2] The World Bank (2013). Fish to 2030: Prospects for Fisheries and Aquaculture.

[3] Bondad-Reantaso M.G., Subasinghe R.P., Arthur J.R., Ogawa K., Chinabut S., Adlard R. (2005). Disease and health management in Asian aquaculture. Vet. Parasitol..

[4] Ernst I., Whittington I.D., Corneillie S., Talbot C. (2002). Monogenean parasites in sea-cage aquaculture. Austasia Aquacult..

[5] Subasinghe R.P. (2005). Epidemiological approach to aquatic animal health management: opportunities and challenges for developing countries to increase aquatic production through aquaculture. Prev. Vet. Med..

[6] Meyer F.P. (1991). Aquaculture disease and health management. J. Anim. Sci..

[7] Defoirdt T., Sorgeloos P., Bossier P. (2011). Alternatives to antibiotics for the control of bacterial disease in aquaculture. Curr. Opin. Microbiol..

[8] Rodger H.D., Adams A. (2016). Fish disease causing economic impact in global aquaculture. Fish Vaccines.

[9] Erdal J.I., Reitan L.J. (1992). Immune response and protective immunity after vaccination of Atlantic salmon (*Salmo salar* L.) against furunculosis. Fish Shellfish Immunol..

[10] Rimstad E., Gudding R., Lillehaug A., Evensen Ø (2014). Vaccination against infectious pancreatic necrosis. Fish Vaccin.

[11] Whelan K. (2010). A Review of the Impacts of the salmon Louse, Lepeophtheirus salmonis (Krøyer, 1837) on Wild Salmonids.

[12] Shinn A.P., Pratoomyot J., Bron J.E., Paladini G., Brooker E.E., Brooker A.J. (2015). Economic costs of protistan and metazoan parasites to global mariculture. Parasitology.

[13] Costello M.J. (2009). The global economic cost of sea lice to the salmonid farming industry. J. Fish. Dis..

[14] Fast M.D. (2014). Fish immune responses to parasitic copepod (namely sea lice) infection. Dev. Comp. Immunol..

[15] Ruane N.M., Jones S.R.M. (2013). Amoebic gill disease (AGD) of farmed Atlantic salmon (*Salmo salar* L.). ICES Identif. Leafl. Dis. Parasites Fish Shellfish.

[16] Adams M.B., Crosbie P.B.B., Nowak B.F. (2012). Preliminary success using hydrogen peroxide to treat Atlantic salmon, *Salmo salar* L., affected with experimentally induced amoebic gill disease (AGD). J. Fish. Dis..

[17] Yanong R.P.E. (2014). Use of hydrogen peroxide in finfish aquaculture. IFAS Ext. Univ. Florida.

[18] Helgesen K.O., Romstad H., Aaen S.M., Horsberg T.E. (2015). First report of reduced sensitivity towards hydrogen peroxide found in the salmon louse *Lepeophtheirus salmonis* in Norway. Aquac. Reports.

[19] Kiemer M.C.B., Black K.D. (1997). The effects of hydrogen peroxide on the gill tissues of Atlantic salmon, *Salmo salar* L.. Aquaculture.

[20] Rodger H.D. (2014). Amoebic gill disease (AGD) in farmed salmon (*Salmo salar*) in Europe. Fish Vet. J..

[21] Treasurer J.W., Grant A. (1997). The efficacy of hydrogen peroxide for the treatment of farmed atlantic salmon, *Salmo salar* L. infested with sea lice (Copepoda: caligidae). Aquaculture.

[22] Burridge L., Weis J.S., Cabello F., Pizarro J., Bostick K. (2010). Chemical use in salmon aquaculture: a review of current practices and possible environmental effects. Aquaculture.

[23] Arvin E., Pedersen L.-F. (2015). Hydrogen peroxide decomposition kinetics in aquaculture water. Aquacult. Eng..

[24] Lyons M.C., Wong D.K.H., Page F.H. (2014). Degradation of Hydrogen Peroxide in Seawater Using the Anti-sea Louse Formulation Interox ®.

[25] Vera L.M., Migaud H. (2016). Hydrogen peroxide treatment in Atlantic salmon induces stress and detoxification response in a daily manner. Chronobiol. Int..

[26] Bowers J.M., Speare D.J., Burka J.F. (2002). The effects of hydrogen peroxide on the stress response of Atlantic Salmon (*Salmo salar*). J. Vet. Pharmacol. Therapeut..

[27] Benfey T.J. (2015). Effectiveness of triploidy as a management tool for reproductive containment of farmed fish: atlantic salmon (*Salmo salar*) as a case study. Rev. Aquacult..

[28] Sadler J., Pankhurst P.M., King H.R. (2001). High prevalence of skeletal deformity and reduced gill surface area in triploid Atlantic salmon (*Salmo salar* L.). Aquaculture.

[29] Withler R.E., Beacham T.D., Solar I.I., Donaldson E.M. (1995). Freshwater growth, smolting, and marine survival and growth of diploid and triploid coho salmon (*Oncorhynchus kisutch*). Aquaculture.

[30] O'Flynn F.M., McGeachy S.A., Friars G.W., Benfey T.J., Bailey J.K. (1997). Comparisons of cultured triploid and diploid Atlantic salmon (*Salmo salar* L.). ICES J. Mar. Sci..

[31] McGeachy S.A., Benfey T.J., Friars G.W. (1995). Freshwater performance of triploid Atlantic salmon (*Salmo salar*) in New Brunswick aquaculture. Aquaculture.

[32] Taylor J.F., Preston A.C., Guy D., Migaud H. (2011). Ploidy effects on hatchery survival, deformities, and performance in Atlantic salmon (*Salmo salar*). Aquaculture.

[33] Taylor J.F., Sambraus F., Mota-Velasco J., Guy D.R., Hamilton A., Hunter D. (2013). Ploidy and family effects on Atlantic salmon (*Salmo salar*) growth, deformity and harvest quality during a full commercial production cycle. Aquaculture.

[34] Taylor J.F., Waagbø R., Diez-Padrisa M., Campbell P., Walton J., Hunter D. (2015). Adult triploid Atlantic salmon (*Salmo salar*) have higher dietary histidine requirements to prevent cataract development in seawater. Aquacult. Nutr..

[35] Fjelldal P.G., Hansen T.J., Lock E.-J., Wargelius A., Fraser T.W.K., Sambraus F. (2016). Increased dietary phosphorous prevents vertebral deformities in triploid Atlantic salmon (Salmo salar L.). Aquacult. Nutr..

[36] Taylor J.F., Leclercq E., Preston A.C., Guy D., Migaud H. (2012). Parr-smolt transformation in out-of-season triploid Atlantic salmon (*Salmo salar* L.). Aquaculture.

[37] Fraser T.W.K., Hansen T., Skjæraasen J.E., Mayer I., Sambraus F., Fjelldal P.G. (2013). The effect of triploidy on the culture performance, deformity prevalence, and heart morphology in Atlantic salmon. Aquaculture.

[38] Fraser T.W.K., Hansen T., Fleming M.S., Fjelldal P.G. (2015). The prevalence of vertebral deformities is increased with higher egg incubation temperatures and triploidy in Atlantic salmon *Salmo salar* L. J. Fish. Dis..

[39] Chalmers L., Taylor J.F., Roy W., Preston A.C., Migaud H., Adams A. (2017). A comparison of disease susceptibility and innate immune response between diploid and triploid Atlantic salmon (Salmo salar) siblings following experimental infection with Neoparamoeba perurans, causative agent of amoebic gill disease. Parasitology.

[40] Chalmers L., Thompson K.D., Taylor J.F., Black S., Migaud H., North B. (2016). A comparison of the response of diploid and triploid Atlantic salmon (*Salmo salar*) siblings to a commercial furunculosis vaccine and subsequent experimental infection with *Aeromonas salmonicida*. Fish Shellfish Immunol..

[41] Moore L.J., Nilsen T.O., Jarungsriapisit J., Fjelldal P.G., Stefansson S.O., Taranger G.L. (2017). Triploid atlantic salmon (*Salmo salar* L.) post-smolts accumulate prevalence more slowly than diploid salmon following bath challenge with salmonid alphavirus subtype 3. PLoS One.

[42] Herath T.K., Ashby A.J., Jayasuriya N.S., Bron J.E., Taylor J.F., Adams A. (2017). Impact of Salmonid alphavirus infection in diploid and triploid Atlantic salmon (*Salmo salar* L.) fry. PLoS One.

[43] Frenzl B., Migaud H., Fjelldal P.G., Shinn A.P., Taylor J.F., Richards R.H. (2014). Triploid and diploid Atlantic salmon show similar susceptibility to infection with salmon lice *Lepeophtheirus salmonis*. Pest Manag. Sci..

[44] Preston A.C., Taylor J.F., Fjelldal P.G., Hansen T., Migaud H. (2017). Effects of temperature on feed intake and plasma chemistry after exhaustive exercise in triploid brown trout (Salmo trutta L). Fish Physiol. Biochem..

[45] Sambraus F., Olsen R.E., Remen M., Hansen T.J., Torgersen T., Fjelldal P.G. (2017). Water temperature and oxygen: the effect of triploidy on performance and metabolism in farmed Atlantic salmon (*Salmo salar* L.) post-smolts. Aquaculture.

[46] Oxley A., Jolly C., Eide T., Jordal A.-E.O., Svardal A., Olsen R.-E. (2010). The combined impact of plant-derived dietary ingredients and acute stress on the intestinal arachidonic acid cascade in Atlantic salmon (*Salmo salar*). Br. J. Nutr..

[47] Fraser T.W.K., Fjelldal P.G., Hansen T.J., Oppedal F., Olsen R.E., Vågseth T. (2014). Aplasia of the septum transversum has no effect on plasma biochemistry following an acute hypoxic event in Atlantic salmon. Dis. Aquat. Org..

[48] Morgan A.L., Thompson K.D., Auchinachie N.A., Migaud H. (2008). The effect of seasonality on normal haematological and innate immune parameters of rainbow trout *Oncorhynchus mykiss* L. Fish Shellfish Immunol..

[49] Betancor M.B., McStay E., Minghetti M., Migaud H., Tocher D.R., Davie A. (2014). Daily rhythms in expression of genes of hepatic lipid metabolism in Atlantic salmon (*Salmo salar* L.). PLoS One.

[50] McStay E., Migaud H., Vera L.M., Sánchez-Vázquez F.J., Davie A. (2014). Comparative study of pineal clock gene and AANAT2 expression in relation to melatonin synthesis in Atlantic salmon (*Salmo salar*) and European seabass (*Dicentrarchus labrax*). Comp. Biochem. Physiol. Mol. Integr. Physiol..

[51] Olsvik P.A., Ørnsrud R., Lunestad B.T., Steine N., Fredriksen B.N. (2014). Transcriptional responses in Atlantic salmon (*Salmo salar*) exposed to deltamethrin, alone or in combination with azamethiphos. Comp. Biochem. Physiol. C Toxicol. Pharmacol..

[52] Untergasser A., Cutcutache I., Koressaar T., Ye J., Faircloth B.C., Remm M. (2012). Primer3-new capabilities and interfaces. Nucleic Acids Res..

[53] Pfaffl M.W. (2001). A new mathematical model for relative quantification in real-time RT-PCR. Nucleic Acids Res..

[54] Pfaffl M.W., Bustin S.A. (2004). Quantification strategies in real-time PCR. A-Z Quant. PCR.

[55] Xie F., Xiao P., Chen D., Xu L., Zhang B., miRDeepFinder (2012). A miRNA analysis tool for deep sequencing of plant small RNAs. Plant Mol. Biol..

[56] Pickering A.D., Pottinger T.G., Hochachka P.W., Mommsen T.P. (1995). Biochemical effects of stress. Biochem. Mol. Biol. Fishes.

[57] Barton B.A. (2002). Stress in fishes: a diversity of responses with particular reference to changes in circulating corticosteroids. Integr. Comp. Biol..

[58] Olsen Y.A., Falk K., Reite O.B. (1992). Cortisol and lactate levels in Atlantic salmon *Salmo salar* developing infectious anaemia (ISA). Dis. Aquat. Org..

[59] Mommsen T.P., Vijayan M.M., Moon T.W. (1999). Cortisol in teleosts: dynamics, mechanisms of action, and metabolic regulation. Rev. Fish Biol. Fish..

[60] Sopinka N.M., Donaldson M.R., O'Connor C.M., Suski C.D., Cooke S.J., Schreck C.B., Tort L., Farrel A.P., Brauner C.J. (2016). Stress indicators in fish. Fish Physiol.

[61] Roque A., Yildiz H.Y., Carazo I., Duncan N. (2010). Physiological stress responses of sea bass (*Dicentrarchus labrax*) to hydrogen peroxide (H2O2) exposure. Aquaculture.

[62] Sanchez J.G., Speare D.J., Johnson G.J., Horney B.S. (1997). Evaluation of the stress response in healthy juvenile rainbow trout after repetitive intermittent treatment with chloramine-T or formalin. J. Aquat. Anim. Health.

[63] Williams H.A., Wootten R. (1981). Some effects of therapeutic levels of formalin and copper sulphate on blood parameters in rainbow trout. Aquaculture.

[64] Benfey T.J., Biron M. (2000). Acute stress response in triploid rainbow trout (*Oncorhynchus mykiss*) and brook trout (*Salvelinus fontinalis*). Aquaculture.

[65] Sadler J., Pankhurst N.W., Pankhurst P.M., King H. (2000). Physiological stress responses to confinement in diploid and triploid Atlantic salmon. J. Fish. Biol..

[66] Sadler J., Wells R.M.G., Pankhurst P.M., Pankhurst N.W. (2000). Blood oxygen transport, rheology and haematological responses to confinement stress in diploid and triploid Atlantic salmon, *Salmo salar*. Aquaculture.

[67] Fraser T.W.K., Mayer I., Skjæraasen J.E., Hansen T., Fjelldal P.G. (2014). The effect of triploidy on the efficacy and physiological response to anesthesia with MS 222 and isoeugenol in Atlantic salmon post-smolts. Aquacult. Int..

[68] Leggatt R.A., Scheer K.W., Afonso L.O.B., Iwama G.K. (2006). Triploid and diploid rainbow trout do not differ in their stress response to transportation. N. Am. J. Aquacult..

[69] Taylor J.F., Needham M.P., North B.P., Morgan A., Thompson K., Migaud H. (2007). The influence of ploidy on saltwater adaptation, acute stress response and immune function following seawater transfer in non-smolting rainbow trout. Gen. Comp. Endocrinol..

[70] Martinez-Porchas M., Martinez-Cordova L.R., Ramos-Enriquez R. (2009). Cortisol and glucose: reliable indicators of fish stress?. Pan Am. J. Aquat. Sci..

[71] Iversen M., Finstad B., McKinley R.S., Eliassen R.A. (2003). The efficacy of metomidate, clove oil, Aqui-S^TM^ and Benzoak® as anaesthetics in Atlantic salmon (*Salmo salar* L.) smolts, and their potential stress-reducing capacity. Aquaculture.

[72] Garcia-Alvarez M., Marik P., Bellomo R. (2014). Stress hyperlactataemia: present understanding and controversy. Lancet Diabetes Endocrinol.

[73] Hirazawa N., Hagiwara H., Tsubone S., Takano R. (2017). Investigation of the toxicological and histopathological effects of hydrogen peroxide bath treatments at different concentrations on *Seriola* species and the effectiveness of these treatments on Neobenedenia girellae (Monogenea) infestations. Aquaculture.

[74] Saurabh S., Sahoo P.K. (2008). Lysozyme: an important defence molecule of fish innate immune system. Aquacult. Res..

[75] Easy R.H., Ross N.W. (2010). Changes in Atlantic salmon *Salmo salar* mucus components following short- and long-term handling stress. J. Fish. Biol..

[76] Caipang C.M.A., Berg I., Brinchmann M.F., Kiron V. (2009). Short-term crowding stress in Atlantic cod, Gadus morhua L. modulates the humoral immune response. Aquaculture.

[77] Fevolden S.E., Roed K.H., Gjerde B. (1994). Genetic components of post-stress cortisol and lysozyme activity in Atlantic salmon; correlations to disease resistance. Fish Shellfish Immunol..

[78] Yildiz H.Y. (2006). Plasma lysozyme levels and secondary stress response in rainbow trout, *Oncorhynchus mykiss* (Walbaum) after exposure to Leteux-Meyer mixture. Turk. J. Vet. Anim. Sci..

[79] Thomassen J.M., Reinertsen H., Dahle L.A., Jorgensen L., Tvinnereim K. (1993). A new method for control of salmon lice. Fish Farming Technol.

[80] Groner M.L., Gettinby G., Stormoen M., Revie C.W., Cox R. (2014). Modelling the impact of temperature-induced life history plasticity and mate limitation on the epidemic potential of a marine ectoparasite. PLoS One.

[81] Aaen S.M., Helgesen K.O., Bakke M.J., Kaur K., Horsberg T.E. (2015). Drug resistance in sea lice: a threat to salmonid aquaculture. Trends Parasitol..

[82] Treasurer J.W., Wadsworth S., Grant A. (2000). Resistance of sea lice, Lepeophtheirus salmonis (Kroyer), to hydrogen peroxide on farmed Atlantic salmon, Salmo salar L. Aquacult. Res..

[83] Bruno D.W., Raynard R.S. (1994). Studies on the use of hydrogen peroxide as a method for the control of sea lice on Atlantic salmon. Aquacult. Int..

[84] Atkins M.E., Benfey T.J. (2008). Effect of acclimation temperature on routine metabolic rate in triploid salmonids. Comp. Biochem. Physiol. Part a - Mol. Integr. Physiol.

[85] Ching B., Jamieson S., Heath J.W., Heath D.D., Hubberstey A. (2010). Transcriptional differences between triploid and diploid Chinook salmon (*Oncorhynchus tshawytscha*) during live *Vibrio anguillarum* challenge. Heredity.

[86] Shrimpton J.M., Sentlinger A.M.C., Heath J.W., Devlin R.H., Heath D.D. (2007). Biochemical and molecular differences in diploid and triploid ocean-type chinook salmon (*Oncorhynchus tshawytscha*) smolts. Fish Physiol. Biochem..

[87] Pala I., Coelho M.M., Schartl M. (2008). Dosage compensation by gene-copy silencing in a triploid hybrid fish. Curr. Biol..

[88] Cleveland B.M., Weber G.M. (2014). Ploidy effects on genes regulating growth mechanisms during fasting and refeeding in juvenile rainbow trout (*Oncorhynchus mykiss*). Mol. Cell. Endocrinol..

[89] Correa K., Filp M., Cisterna D., Cabrejos M.E., Gallardo-Escárate C., Yáñez J.M. (2015). Effect of triploidy in the expression of immune-related genes in coho salmon *Oncorhynchus kisutch* (Walbaum) infected with *Piscirickettsia salmonis*. Aquacult. Res..

[90] Ren L., Tang C., Li W., Cui J., Tan X., Xiong Y. (2017). Determination of dosage compensation and comparison of gene expression in a triploid hybrid fish. BMC Genom..

[91] Tort L. (2011). Stress and immune modulation in fish. Dev. Comp. Immunol..

[92] Saeij J.P., Verburg-van Kemenade L.B., van Muiswinkel W.B., Wiegertjes G.F. (2003). Daily handling stress reduces resistance of carp to *Trypanoplasma borreli*: in vitro modulatory effects of cortisol on leukocyte function and apoptosis. Dev. Comp. Immunol..

[93] Nardocci G., Navarro C., Cortés P.P., Imarai M., Montoya M., Valenzuela B. (2014). Neuroendocrine mechanisms for immune system regulation during stress in fish. Fish Shellfish Immunol..

[94] Tort M.J., Hurley D., Fernandez-Cobas C., Wooster G.A., Bowser P.R. (2005). Effects of hydrogen peroxide treatments on catalase and gluthathione activity in walleye *Sander vitreus*. J. World Aquacult. Soc..

[95] Fast M.D., Johnson S.C., Jones S.R.M. (2007). Differential expression of the pro-inflammatory cytokines IL-1β-1, TNFα-1 and IL-8 in vaccinated pink (*Oncorhynchus gorbuscha*) and chum (*Oncorhynchus keta*) salmon juveniles. Fish Shellfish Immunol..

[96] Fast M.D., Hosoya S., Johnson S.C., Afonso L.O.B. (2008). Cortisol response and immune-related effects of Atlantic salmon (*Salmo salar* Linnaeus) subjected to short- and long-term stress. Fish Shellfish Immunol..

[97] Metz J.R., Huising M.O., Leon K., Verburg-van Kemenade B.M.L., Flik G. (2006). Central and peripheral interleukin-1b and interleukin-1 receptor I expression and their role in the acute stress response of common carp, *Cyprinus carpio* L. J. Endocrinol..

[98] Velkova-Jordanoska L., Kostoski G., Jordanoska B. (2008). Antioxidative enzymes in fish as biochemical indicators of aquatic pollution. Bulg. J. Agric. Sci..

[99] Kumari K., Khare A., Dange S. (2014). The applicability of oxidative stress biomarkers in assessing chromium induced toxicity in the fish *Labeo rohita*. BioMed Res. Int..

[100] Perry J.J.P., Shin D.S., Getzoff E.D., Tainer J.A. (2010). The structural biochemistry of the superoxide dismutases. Biochim. Biophys. Acta Protein Proteonomics.

[101] Duracková Z., Gvozdjáková A. (2008). Oxidants, antioxidants and oxidative stress. Mitochondrial Med.

[102] Langston A.L., Johnstone R., Ellis A.E. (2001). The kinetics of the hypoferraemic response and changes in levels of alternative complement activity in diploid and triploid Atlantic salmon, following injection of lipopolysaccharide. Fish Shellfish Immunol..

