# Supplementary material for: Comparative ploidy response to experimental hydrogen peroxide exposure in Atlantic salmon (Salmo salar)
Source: Fish Shellfish Immunol. 2018 Oct;81:354–67. doi: 10.1016/j.fsi.2018.07.017 (PMC6115329; doi:10.1016/j.fsi.2018.07.017)
Supplement: Supplement1 - Efficiency table [file mmc1.docx]

Supplementary table 1: Efficiencies (%) of the real-time PCR assays for all genes assessed in liver and gill tissue from diploid and triploid Atlantic salmon.

|  | DIPLOID | | TRIPLOID | |
| --- | --- | --- | --- | --- |
| Gene | LIVER | GILL | LIVER | GILL |
| β-actin | 88.4 | 96.2 | 95 | 95.4 |
| ef1α | 94.7 | 98.6 | 99 | 90.1 |
| rpl1 | 91.9 | 104 | 104 | 94.8 |
| rpl2 | 97.1 | 99.9 | 98 | 94.7 |
| b2m | 84.3 | 88.5 | 88.2 | 90 |
| cat | 87.6 | 97.6 | 88.2 | 92.6 |
| gpx1 | 96.7 | 95.8 | 108 | 95.6 |
| gr | 102 | 98.9 | 104 | 95.6 |
| hsp70 | 97.1 | 100 | 93.4 | 90.8 |
| sod1 | 91.4 | 93.7 | 87.3 | 96.2 |
| sod2 | 92.1 | 89.4 | 88 | 92.3 |
| saa5 | 85.9 | 98.6 | 78.2 | 87.4 |
| crp/sap1a | 94.1 | 96.2 | 92.2 | 70.6 |
| crp/sap1b | 88.5 | 91.6 | 91.9 | 89.8 |
| il1β | N/A | 95.9 | N/A | 98.5 |
